# Infection and Contagion

**Published:** 1854-02

**Authors:** 


*Infection and Contagion.*—We find in the *Revue Therapeutique du Midi,* the organ of the once celebrated, and still highly respectable, school of Montpellier, the following judicious remarks upon the difference between infection and contagion, from the pen of Dr. Saurel, editor-in-chief of that excellent periodical.

" I desire to present to my readers what I consider the true medical doctrines in regard to infection and contagion, by briefly analyzing the opinions advanced by M. Anglada, in his work on contagion, and by M. Jaumes, in his course on pathology and general therapeutics.

By the term *infection,* we designate the morbid action, that air, impregnated with certain heterogeneous principles of organic and non-virulent origin, produces on a healthy subject. We cannot give the name infection to the morbific effects of air charged with inorganic principles, such as arsenic, mercury, carbonic acid, etc.; in these cases there may be poisoning, but there is no infection. Nor is this epithet more appropriate to the effects of air charged with heterogeneous virulent principles; under these circumstances contagion and not infection is the result.

The effluvia of marshes, putrid emanations, and miasmata, constitute three categories of causes of infection, which include all others, and which differ as much in their origin as in their degree of intensity.

The *effluviæ* derived from the decomposition of animal and vegetable matters in the water of swamps, are very volatile; they cannot be detected by our instruments or modes of analysis, and they extend to great distances from the localities in which they originate. The diseases which they produce are diverse in their character ; their effects are rarely confounded with those of contagion.

*Putrid emanations* are principally disengaged from decomposing animal substances; they remain, so to speak, concentrated in the places where they are formed; their effects are most rapid and most pernicious, and very often they constitute the vehicle of contagion.

*Miasms* are of animal origin; they are given off from living bodies, whether healthy or unhealthy, when they are crowded together in a confined atmosphere. Their action is more energetic than that of effluviæ; they produce diseases of a typhoid character; they extend to a small distance only, and their effects resemble those of contagion.

Such are the sources of infectious diseases. It will be observed that although each category occasions more particularly a certain class of morbid effects, there nevertheless exists no direct relation of cause and effect; thus, a miasm, for example, may give rise to typhus, or hospital gangrene, or dysentery, or to other equally distinct diseases; whereas contagion, as we shall see presently, can only occasion a disease similar to the one in which it originates.

What now is contagion? "It is," says M. Anglada, " the transmission of a morbid affection from a sick person to one or many individuals, by the medium of a material principle, which, being the product of a specific morbid elaboration, induces, in those it attacks, either mediately or immediately, a disease similar to that from which it proceeds, provided predisposition exists,"

Contagion cannot be admitted without admitting *virus* also; the virus is, in fact, the material principle which transmits the disease. Every contagious disease, therefore, produces a virus. But if, in variola, syphilis, hydrophobia, the glanders, etc., this is admitted without debate, it is quite different with other species of virus less easily demonstrated. Thus the viruses of plague, of yellow fever, of typhoid fever, of hooping cough, are by no means universally admitted. In these cases the material principle being intangible, and invisible, is denied by men who perceive only infection in what in reality is contagion. If every virus had been concrete, solid, or liquid, the quarrel between the contagionists and the infectionists would have ended long ago. It is solely because many kinds of virus exist in a gaseous or aeriform state, that the contagiousness of certain diseases is still a debatable point. But can the form, or physical condition, of a virus change in any way its mode of action? Water in the state of vapor, however diffused it may be, is it not always water? The virus of small-pox does not cease to be a virus, when instead of being solid, as in crusts, liquid, as in the matter of pustules, it encompasses the patient with a virulent atmosphere. Whether you respire the morbiferous atmosphere in the patient's chamber, or whether you touch the virus with the finger, is there not contact always, is there not contagion?

Thus, in principle, the distinction between infection and contagion is easy: contagion occurs whenever, a virus having been elaborated, there is transmisson by mediate or immediate contact, of a morbid affection from a diseased to a healthy person; in all other cases there is no contagion; there may be infection.

A most important question here arises. Can we establish a class of contagious diseases? Is contagiousness a characteristic which may distinguish certain diseases? We do not hesitate to reply in the negative. It is perfectly true that certain diseases are usually transmitted by contagion, as, for example, syphilis, measles, scarlatina, etc., but there is nothing to prove that these affections may not be spontaneously developed. The diseases which are most contagious must have had a commencement—an origin. The diseases which are most generally contagious are not necessarily so; it is not only requisite that there should be sufficient morbid capacity in a healthy subject in order that he may contract a disease, it is also necessary that the virus elaborated by the sick man should have sufficient activity. If, from any cause, this activity diminishes, contagion will either become rarer, or it will not take place at all; in this way is explained the cessation of contagious diseases. Certain circumstances, on the other hand, such as epidemic influence, and infection more particularly, may lend increased energy to the virus, greater activity to the contagious principle.

We have said that a class of contagious diseases cannot be set apart. Does this mean that all diseases may become contagious? Such a proposition is evidently an exaggeration. There are certain diseases which can never assume this character; nervous diseases are of this number. Although convulsions often occur successively in a great number of persons, there is no contagion in such cases; there are instances of *morbid transmissions by*

*imitation*, to which M. Anglada has devoted an interesting article in his work. Some acute diseases, as pneumonia, hepatitis, and encephalitis, appear insusceptible of assuming a contagious character; but we should be reserved on this point, for other affections, quite as acute and inflammatory, have appeared at times to be transmitted by contagion.

Daily observation teaches that contagiousness is especially developed in those diseases accompanied by an alteration of the fluids; the co-existence of a febrile condition and of abundant excretions seems to favor the development of a virulent principle.

In practice, it is not always easy to distinguish contagion from infection; indeed the two influences are often mingled. The following facts may throw some light on this question. It cannot be denied that certain diseases usually arise from infection, while others are generally produced by contagion. If a great number of individuals are simultaneously or successively attacked by the same disease, in order to discover whether the disease is propagated by contagion or infection, it is sufficient to recall its usual origin. If it is a disease of infectious origin, although it has accidently assumed a contagious character, the indication is not at all modified by this circumstance; it is requisite to destroy the infectious cause, if this is possible,—to purify or evacuate the focus of infection and to disperse the sick, taking precautions against contagion at the same time. If, on the contrary, the disease is usually contagious, the indication is totally different: the isolation of the patients is the first condition to be fulfilled

In these cases the duty of the practitioner is plain. But suppose several healthy individuals enter an hospital in which some infectious disease is prevailing; a short time afterward, some are attacked by dysentery, others by typhoid fever, others by typhus. Among the persons who have contracted typhus, is one, living in a salubrious and well-ventilated locality, far from the centre of infection, whose attendants one after the other contract the same disease. Does the transmission still occur by infection? Certainly not; in this case there is contagion. We are well aware that our explanation of infection in the one case and contagion in the other, may be criticised; but practically, this is of no consequence. Whenever contagion is suspected, we should act as if it existed, and advise precautions. Thus, in practice, infection is distinguished from contagion by producing many distinct diseases, while contagion can only give rise to one disease similar to the one from which it arose.

We wish to say a word before concluding upon another question, namely, the relation of contagion to disease. Many physicians consider contagion a *morbid element*. They term whatever throws light either on the diagnosis or treatment of a malady the *elements* of that malady. Contagion, say the partizans of this opinion, fulfills these two conditions. Contagion might elucidate the diagnosis of a disease if it was constant, and could be produced at will and without danger; but this is not so, and it is well known that the inoculations of which M. Ricord and his followers have made such great abuse, have thrown but little light upon the diagnosis of syphilis, the most contagious of all diseases. For a still stronger reason, contagion will not elucidate those diseases in which it is not an invariable quality.

As to the treatment, when we recognize the contagious nature of a disease, we prescribe what is necessary to prevent its propagation, but this is not therapeutics, but prophylaxis or hygiene. The patient gains nothing by these precautions. What is it to him whether his disease be contagious or no? Does the idea of contagion suggest to us a single remedy, a single method of treatment? Contagion therefore, elucidating neither the diagnosis nor treatment, is not a morbid element. It is a quality—a property of a disease, which it may assume or lose without its other characters being in any way modified.

Therefore, to sum up, let us say with M. Anglada, "that in the majority of diseases, contagion is not a condition *sine qua non* of their nature; that far from being indissolubly associated with them, it is only added as a sort of complication, and that the actions of the economy preserve in these as in all other cases, their independence."—*Virginia Medical & Surgical Journal.*

